# Evaluation of Ethiopia’s field epidemiology training program – frontline: perspectives of implementing partners

**DOI:** 10.1186/s12913-023-09384-w

**Published:** 2023-04-26

**Authors:** Tolcha Kebebew, Mahlet A. Woldetsadik, Jordan Barker, Angelina Cui, Aisha A. Abedi, David E. Sugerman, Desmond E. Williams, Reina M. Turcios-Ruiz, Tariku Takele, Neima Zeynu

**Affiliations:** 1 Division of Global Health Protection, Center for Global Health, US Centers for Disease Control and Prevention, Addis Ababa, Ethiopia; 2 Division of Global Health Protection, Center for Global Health, US Centers for Disease Control and Prevention, Atlanta, GA USA; 3 Oak Ridge Institute for Science and Education (ORISE) Fellow, Oak Ridge Associated Universities, Oak Ridge, TN USA; 4 Center for Public Health Emergency Management, Ethiopian Public Health Institute, Addis Ababa, Ethiopia

**Keywords:** Field Epidemiology, FETP-Frontline, Evaluation, Ethiopia

## Abstract

**Background:**

Field Epidemiology Training Program (FETP) has been adopted as an epidemiology and response capacity building strategy worldwide. FETP-Frontline was introduced in Ethiopia in 2017 as a three-month in-service training. In this study, we evaluated implementing partners’ perspectives with the aim of understanding program effectiveness and identifying challenges and recommendations for improvement.

**Methods:**

A qualitative cross-sectional design was utilized to evaluate Ethiopia’s FETP-Frontline. Using a descriptive phenomenological approach, qualitative data were collected from FETP-Frontline implementing partners, including regional, zonal, and district health offices across Ethiopia. We collected data through in-person key informant interviews, using semi-structured questionnaires. Thematic analysis was conducted, assisted with MAXQDA, while ensuring interrater reliability by using the consistent application of theme categorization. The major themes that emerged were program effectiveness, knowledge and skills differences between trained and untrained officers, program challenges, and recommended actions for improvement. Ethical approval was obtained from the Ethiopian Public Health Institute. Informed written consent was obtained from all participants, and confidentiality of the data was maintained throughout.

**Results:**

A total of 41 interviews were conducted with key informants from FETP-Frontline implementing partners. The regional and zonal level experts and mentors had a Master of Public Health (MPH), whereas district health managers were Bachelor of Science (BSc) holders. Majority of the respondents reflected a positive perception towards FETP-Frontline. Regional and zonal officers as well as mentors mentioned that there were observable performance differences between trained and untrained district surveillance officers. They also identified various challenges including lack of resources for transportation, budget constraints for field projects, inadequate mentorship, high staff turnover, limited number of staff at the district level, lack of continued support from stakeholders, and the need for refresher training for FETP-Frontline graduates.

**Conclusions:**

Implementing partners reflected a positive perception towards FETP-Frontline in Ethiopia. In addition to scaling-up the program to reach all districts to achieve the International Health Regulation 2005 goals, the program also needs to consider addressing immediate challenges, primarily lack of resources and poor mentorship. Continued monitoring of the program, refresher training, and career path development could improve retention of the trained workforce.

## Introduction

Workforce for health is central to the delivery of adequate and quality health services. The Workforce 2030 strategy of the World Health Organization (WHO) promotes strengthening countries’ workforce capacity to prevent, detect, and respond to emergencies and disasters [1]. The International Health Regulation (IHR) 2005 also encourages member states of the WHO to fulfill workforce development core capacity and achieve effective prevention and control of public health emergencies of national and international concern [2].

The Field Epidemiology Training Program (FETP) is an interactive training adapted from the US Epidemic Intelligence Services [3]. The training was initially adopted in some countries, including Canada [4], Colombia [5], Germany [6, 7], South Korea [8], and other Central and Southern American countries [9]. The IHR 2005 and its subsequent evaluations have promoted FETP as a milestone of the health workforce for public health emergency management (PHEM) [2, 10, 11]. Following IHR 2005, over 80 countries worldwide, including Ethiopia, have adopted the program to build epidemiology and response capacity [12].

FETP was developed as a three-tier interlinked career path, from frontline to intermediate and advanced [13]. It is designed to train a large number of the health workforce through the frontline (lowest level, commonly district-based) and the intermediate (region and sub-region based) programs. The highest level, FETP-Advanced, was designed for national and regional level health professionals. The FETP curriculum consists of minimal classwork-based deductive education and over 70% practical exercise at the field sites.

**Fig. 1:**
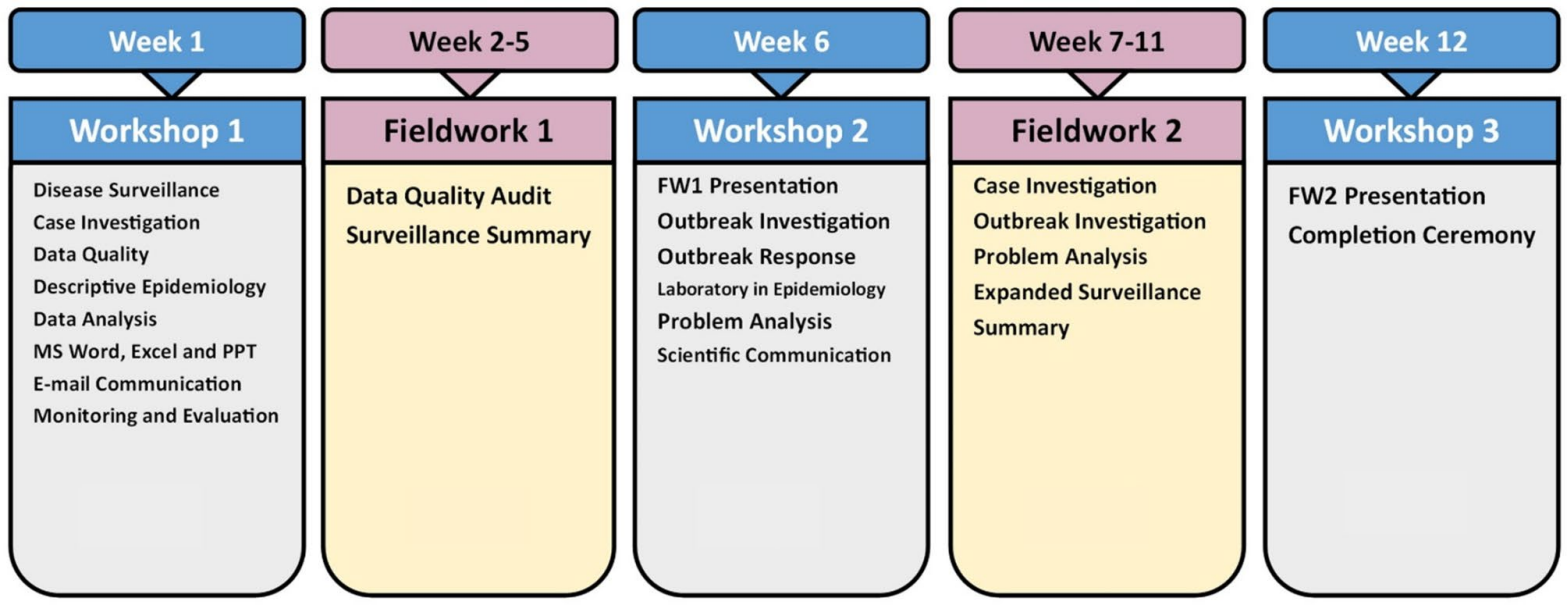
Ethiopia’s curriculum for the Field Epidemiology Training Program-Frontline, [Adapted from the Centers for Disease Control and Prevention]. Note: Abbreviations – FW1: Field Work-1; FW2: Field Work-2; the figure has also been published elsewhere [16]

Ethiopia adopted FETP- Frontline in 2017, hosted by the Ethiopian Public Health Institute (EPHI) with technical and financial support from the US Centers for Disease Control and Prevention (CDC). The curriculum was adapted from CDC’s Frontline FETP guide [14, 15] with some modifications (Fig. 1). The training includes three workshops and two fieldwork projects. The core contents of the training during the workshops include: basic epidemiology; descriptive statistics; data management and analysis using Microsoft Excel; reporting and presentation of surveillance information using Microsoft Word and PowerPoint; and monitoring and evaluation. The fieldwork projects focus on surveillance data quality audit; analysis and presentation of data; case investigations; analysis of a root cause of surveillance problems; and outbreak investigations.

FETP-Frontline aims to build the capacity of district-level surveillance officers to prevent, detect, and respond to public health emergencies. Implementing partners include regional health bureaus, zonal health departments, and district health offices. The surveillance department of the regional health bureau and zonal health departments play key roles in the selection of training participants, facilitation of the workshops, and provision of mentorship during the fieldwork activities. The support provided by the district health office to the trainees includes provision of logistics and materials necessary for project activities.

By the end of March 2019, the program had successfully trained over 350 surveillance officers across all regions and city administrations in Ethiopia. In late 2019, EPHI in collaboration with its partners, initiated an evaluation to assess the effectiveness of FETP-Frontline in improving surveillance officers’  knowledge, skills, and practices and public health emergency response activities. The evaluation proposal included both quantitative assessment of skills and knowledge of the surveillance officers [16] and qualitative assessment of perception of implementing partners towards the program. This manuscript is focused on a qualitative assessment of perceptions of regional and zonal surveillance officers, mentors, and district health managers towards the effectiveness of FETP-Frontline and also aimed to identify challenges and recommendations for the program’s improvement.

## Methods

### Design

In this study, we utilized a qualitative cross-sectional study to evaluate FETP-Frontline in Ethiopia. A descriptive phenomenology approach was followed to explore the perception of implementing partners towards the program and to identify program challenges and recommendations. We conducted key-informant interviews with experts from implementing partners, including regional, zonal, and district health offices.

### Research sites and sample

The study was conducted in Addis Ababa City Administration and five regions in Ethiopia – Amhara, Afar, Oromia, Southern Nations Nationalities and Peoples’ Region (SNNPR), and Tigray. These regions have FETP-Frontline fully implemented. From these regions, sub-regions (called *Zones*) and districts were randomly selected for the study. The full selection methodology has been described elsewhere [16]. From the selected zones, leads of surveillance teams and mentors were interviewed. For all selected districts, the health office heads, who are supervisors of FETP-Frontline graduates, were interviewed.

### Data collection

Ten trained interviewers from EPHI collected the data. The interviews were conducted in three regional local languages (Amharic, Afaan Oromo, and Tigrigna) using a semi-structured interview guide developed and pretested for this study. The interview guide focused on assessing perceptions towards FETP-Frontline; FETP-Frontline’s impact on surveillance and emergency management; and performance differences among districts with trained and untrained officers. Participants also identified challenges during the program implementation and recommendations.

### Data management and analysis

All interviews were audio recorded, transcribed verbatim, and translated from the local languages to English by trained data collectors. Data were uploaded into MAXQDA Version 20.0.2, a mixed-methods analysis software program, which facilitated team-based coding and subsequent data analysis. We used an inductive and deductive approach to develop a codebook that accounted for key research questions covered by the interview protocol as well as allowed for the emergence of novel themes during coding and analysis. The final codebook represented the key domains of interest to this evaluation (e.g., mentorship, perception towards FETP-Frontline, differences between trained and untrained surveillance officers, and recommendations for improving the program) and themes provided substantive patterns and examples within these domains.

Three team members (MAW, JB, and AC) experienced in qualitative data analysis coded the interviews. The first set of the coding scheme was used by JB and AC to code four transcripts independently. This was then checked by MAW, refined, and expanded with the analytic team. To ensure inter-rater reliability, the team reviewed a random selection of transcripts to ensure consistent application of theme categorizations. The coding team met frequently over the course of May and June 2021 to resolve any discrepancies in the application of the codes. This process established inter-coder reliability, including consistent application of codes within the MAXQDA platform. Upon the completion of coding, the team reviewed the coded excerpts to identify themes through well-established techniques, such as repetition, if a theme was expressed multiple times, and comparing within and across the four types of partners for similarities and differences in their perception of FETP-Frontline in Ethiopia [17].

### Ethical considerations

This evaluation received ethical clearance from the Ethiopian Public Health Institute’s Institutional Review Board (Protocol #: EPHI-IRB-205-2019) and permission was solicited from all regions where the study was conducted. All respondents provided written consent and their right to decline participation at any point during the interview was explained to them.

## Results

### Participant characteristics

We conducted a total of 41 interviews. These included six regional health bureau PHEM officers, seven zonal (sub-regional) health department PHEM officers, eight mentors, and 20 heads of district health offices (Table 1). Regional and zonal PHEM officers as well as mentors had Master of Public Health in Field Epidemiology whereas district health managers had bachelor’s degrees. In addition to their overall perception regarding the training program, regional and zonal PHEM officers and mentors shared their views on performance differences among the trained and untrained district surveillance officers.

**Table 1:** Characteristics of respondents in the Ethiopia Field Epidemiology Training Program (FETP) Frontline evaluation, September 2019

Respondents’ Characteristics (n = 41)	Number (%)
**Participant type**	
Head of District Health Office	20 (48.8)
Mentor	8 (19.5)
Zonal (sub-regional) Health Department PHEM^a^ Officer	7 (17.1)
Regional Health Bureau PHEM Officer	6 (14.6)
**Education**	
Bachelor of Science	20 (48.8)
Master of Public Health	21 (51.2)
**FETP-Frontline Status**	
Supervisor of FETP-Frontline Graduates and Non-trained	21 (51.2)
Health Manager of District with FETP-Frontline Graduates	20 (48.8)
**Region**	
Oromia	11 (26.8)
Amhara	9 (22)
SNNPR^b^	8 (19.5)
Tigray	6 (14.6)
Afar	5 (12.2)
Addis Ababa	2 (4.9)

^a^*PHEM* Public Health Emergency Management, ^b^*SNNPR* Southern Nations, Nationalities and Peoples’ Region

### Respondents’ perception of FETP-Frontline

Majority of the respondents reflected a positive perception towards the FETP-Frontline program. Most respondents noted that they observed improvements in the knowledge and skills of FETP-Frontline graduates. A few respondents discussed improvements between FETP-Frontline trained and untrained officers but noted that trained surveillance officers could not fully demonstrate their increased set of skills due to barriers affecting response activities in their community, such as budget issues.

*“The training [FETP-Frontline] has made the woredas to develop performance capacity, … In addition, it enabled them to show improvement to use data for informed decision-making process through data analysis and interpretation.”* Regional Health Bureau, Southern Nations, Nationalities and Peoples’ Region

### FETP-Frontline increased the skills of PHEM

Most respondents commented on the improved skills of FETP-Frontline graduates. Respondents noted increased engagement among staff, greater understanding of their position as a surveillance officer, and increased knowledge of response to public health emergencies. Two respondents clearly noted that their communities are now more closely involved with emergency response and support health officials by reporting potential cases.

#### Improvements in case and outbreak investigations and responses

Respondents shared that FETP-Frontline has enhanced early detection and response to public health emergencies. Two respondents provided specific details on recent outbreaks in their communities that were quickly identified and controlled due to the trainees’ increased knowledge post-training. Another respondent described an improved focus on emergency response in their community.

*“First of all, it [FETP-Frontline] increased the awareness of the PHEM focal person [FETP-Frontline trainee] on how to respond to different emergencies”* Woreda Health Office, Oromia region.

*“FETP [Frontline] has contributed to early detection and response in our woreda, for example, our woreda is affected by Malaria so the number of malaria cases were increasing seasonally. Because of FETP [Frontline], cases were detected early and response activities such as residual spray interventions were undertaken promptly.”* Woreda Health Office, Southern Nations, Nationalities and Peoples’ Region.

#### FETP-Frontline improved data quality and reporting

Some respondents emphasized that there were noticeable positive changes in the quality of data and reporting methods after surveillance officers graduated from FETP-Frontline. Several comments included specific examples of improvements such as enhanced surveillance methods in addition to completed reports submitted to and from health facilities.

*“Surveillance data quality was improved at health facility and at the health office level … completeness of reported data from health post was improved”* Woreda? Health Office, Oromia region.

#### FETP-Frontline trainees shared lessons learned

Lessons learned from trainees of FETP-Frontline were also discussed by the respondents. They mentioned how trained officers provided valuable contributions to their communities through increased awareness of emergency response protocols for fellow healthcare professionals, capacity building, reporting systems, formatting, and assistance in training other healthcare staff. As a result of shared lessons learned, one respondent discussed how the increased awareness between properly trained healthcare staff and community members aided in reducing malaria cases in their community.

*“...malaria disease burden was high; however, the trend has decreased from year to year. Environmental control activities were widely performed. Malaria outbreaks have decreased as a result of trained health care professionals who supported lower-levels, and the lower-level focal persons mobilized the community and performed activities together with health extension workers.”* Woreda Health Office, Amhara region.

### Differences between trained and untrained surveillance officers

Most respondents mentioned differences in surveillance performance between surveillance officers who are trained in FETP-Frontline and those who are untrained. There were two themes that arose from the discussion: (1) differences in data collection, analysis, scientific reporting, and communication; and (2) differences in case and outbreak investigations and response.

#### Data collection, analysis, scientific reporting, and communication

Respondents mentioned how trained and untrained surveillance officers differed in data collection and analysis, scientific reporting, and communication. Trained surveillance officers were able to complete data collection and analysis more thoroughly and effectively communicated results to implementing partners. Respondents observed that before FETP-Frontline, data quality was extremely poor, and after FETP-Frontline there was an improvement in data completeness and consistency. While practical changes were noticeable, some respondents advocated for evaluations to assess changes.

*“There is a great difference between woredas that have taken FETP–Frontline training as compared those who did not have training. Firstly, those woredas with trained personnel effectively collected, analyzed data, and shared it with us. They also display the data in different forms. They could effectively monitor the data generated from health facilities. However, those who did not get the training lacked all the above-mentioned activities. In general, those woredas with trained frontline-field epidemiology program had a good status in conducting PHEM activities as compared with those who had not get training.”* Regional PHEM Officer, Afar Region.

#### Case and outbreak investigations and responses

Respondents also mentioned how there were noticeable differences between trained and untrained surveillance officers in timeliness and completeness of case and outbreak investigations. Untrained surveillance officers were observed to identify outbreaks; however, the trained officers took initiative with more structured public health and emergency management responses.

*“The people who are trained are not the same as those that were not trained. For example, the untrained just tell you (ok there is diarrhea) whereas the one trained goes through the case definitions, the confirmation process, knows who to contact, etc. They follow a structure that helps the surveillance system.”* PHEM Officer, Addis Ababa City Administration Health Bureau.

In all conversations, respondents mentioned how FETP-Frontline training improved surveillance officers’ confidence, understanding of their scope of work, and ability to work independently. Trained surveillance officers were observed to be more confident in completing problem analysis, outbreak investigations, and case investigations independently.

*“It [FETP-Frontline] is a critical and essential program, those who took FETP-Frontline training do the jobs by themselves, not waiting for us. They do problem analysis, outbreak investigation, and case investigation. They know what their role and responsibilities are but those who did not take the training don’t know clearly what their roles and responsibilities are. Since it is on-site training, it helps them to practice what they learn.”* Mentor, Oromia Region.

### Opportunities with FETP-Frontline

#### Benefits of mentorship

Mentors outlined some benefits of mentorship such as gained professional knowledge and experience, staying informed of the latest training workshops, and expressed self-satisfaction of being a mentor. One participant cited that the positive facets of mentorship outweighed the lack of incentives that mentors receive for embracing this position.

*“Regarding incentives, it is not much, if you were with them and saw the trainee’s motivation, you wouldn’t worry about it since you advance your knowledge, learn and update yourself; furthermore, mentorship was expected from us.”* Mentor, South Nations, Nationalities and Peoples’ Region.

#### Professional development/skills building

Mentors described the professional development and skills building opportunities as an overall positive aspect of FETP-Frontline. Overall, respondents agreed that they gained additional knowledge and experience while serving as a mentor to FETP-Frontline trainees.

*“Professional career development, increased knowledge, skill development and in general, improved surveillance.”* Mentor, Amhara region.

### Challenges with FETP-Frontline

Several challenges of FETP-Frontline were identified amongst respondents; primary challenges described included budget issues, difficulties with transportation, shortage of resources, and workforce shortages that affected the implementation of FETP-Frontline training. Some of these challenges also hindered the trainees in applying what they learnt to the surveillance activities. While some respondents only noted a primary challenge in their community, there were several respondents that outlined multiple challenges that are generally affecting the Ethiopian public health system. Resource shortages included water, medications, and emergency healthcare facilities. Other challenges included malnutrition within communities, rough terrain on transportation routes, political instability, high turnover among healthcare staff, lack of trained healthcare staff, and unsatisfactory pay for officers. These challenges affected the overall health system, including surveillance and response to public health emergency management activities.

*“One of our major challenges in relation to this high population density is, there are no enough emergency logistics. There is also no adequate transportation system. We have only one motor bicycle to monitor and help emergency related activities in our woredas. There is no sufficient compensation for the time devoted by our officers to tackle emergency problems. This will hinder our services and prevent a smooth running of our activities.”* Woreda Health Office, Afar region.

#### Budget issues

Most of the respondents outlined budget constraints more than any other challenges faced by Ethiopia’s FETP-Frontline. Despite seeing notable improvements in skills from the FETP-Frontline trainees, one participant believed that the budget issues undermined these improvements and made it difficult for the health system to fully provide the services for the community.

*“However, the improvement is not going as we expected. This is because of some constraint like shortage of budgets. This all hinders the system, and we cannot serve our community as expected.”* Woreda Health Office, Afar region.

Other respondents described their budget as “small”, “insufficient” or “inadequate”. Multiple respondents cited a lack of reliable transportation because of these issues, or associated costs depleting their budget. If public health emergencies arose within these communities, some respondents identified that they had no reserved budget specified for emergency response, and therefore, they extracted funding from other programs to respond to emergencies. As a result, less funding remained for the intended programs for preventive measures, such as vaccinations.

*“The woreda allocated small budget to strengthen its PHEM program. For this program, we specifically allocate budget for anticipated emergency in the future. But it is insufficient. It is very small.”* Woreda Health Office, Amhara region.

#### Lack of monitoring and follow up

Lack of follow up was highlighted as a concern within FETP-Frontline, especially from the federal levels of health systems. One participant stressed the importance of continued capacity development after completing FETP-Frontline, as they saw no improvements in the skills of graduates in response time for public health emergencies. Other respondents cited the need for follow up to further strengthen the surveillance system and prevent delays in emergency response due to issues surrounding budget allocation.

*“Any support provided by the federal government needs follow up. To respond to an emergency, we can’t wait for budget approvals. We are on the front lines and if we are late in responding, people die.”* Woreda Health Office, Oromia region.

#### Mentorship issues

Several mentorship-related challenges within FETP-Frontline were discussed. Numerous mentors cited that there were limited amounts of time to devote to mentee support. Other respondents cited low motivation from mentees or lack of resources required for mentorship such as computers, internet access, and transportation. Additionally, multiple respondents mentioned poor computer skill levels for mentees, which requires additional time and devotion from the mentors.

*“The challenge is resource shortage and logistics. Skills of the trainees on computer are low. I am working alone intensively on that part, and they [mentees] have to know basic computer skill, at least MS Excel, … so that they do analysis. … there is also shortage of other logistics, like paper and other stationeries.”* Mentor, South Nations, Nationalities and Peoples’ Region.

### Recommendations for improving FETP-Frontline

While respondents generally had a positive perception of FETP-Frontline and noted observable differences from trained surveillance officers to untrained surveillance officers, respondents still voiced strong recommendations. Most respondents offered at least one recommendation for improving FETP-Frontline in Ethiopia. Three major themes emerged: (1) Additional trainings should be given, (2) Mentorship support should be strengthened to address challenges faced by mentors; and (3) The Ethiopian government and other organizations, such as the EPHI and CDC, should provide continuous support to FETP.

#### Additional training

Respondents requested for FETP-Frontline to be scaled up to reach other health professionals and staff members in different woredas. Since additional FETP-Frontline training could help offset staff turnover and establish workforce sustainability in the health sector. Respondents also mentioned the need for refresher courses for FETP-Frontline graduates, such as training in computer and statistical software and data management (R, SPSS, Stata, Arc GIS, Quantum GIS, Epi Info).

*“So, the frontline training should address other departments especially disease prevention and health promotion department which works in parallel with PHEM. This can help them to give on-job training to lower level. Refresher training were not provided to those who got the training before. PHEM focal at health centers are new; they didn’t get a chance to attend training, so the training needs to address other health office departments and health center focals.”* Woreda Health Office, Southern Nations and Nationalities and Peoples’ Region.

#### Improve mentorship

Respondents mentioned that mentorship should be strengthened, and mentors should be given sufficient support during FETP-Frontline implementation. This included providing appropriate equipment, hiring enough mentors and support staff, and providing transportation. Mentors stated that they addressed current challenges by working outside of office hours, working without essential equipment, including computer and internet, and utilizing strong communication practices with both FETP-Frontline trainees and political leaders. Supporting mentors sufficiently would help to cascade down training and provide better mentorship for FETP graduates. Mentors also need adequate training on the FETP-Frontline curriculum and deliverables, mentorship principles, leadership, and supervisory skill to facilitate learning and knowledge transfer.

*[What could have helped you address these challenges?] “Oh, support. On-the-job support can be included if it is needed. Payment and facilitating transportation are needed to do on-the-job support. Time is also to be considered. Mentor and mentee are to be free from working load during mentorship … Especially, mentees should be free from responsibilities to perform their outputs. Enough time should be provided to them. There should be enough payment.”* Mentor, Amhara Region.

#### Continuous support from ethiopian government and stakeholders

Respondents recommended that continuous support be provided from the Ethiopian government, partners, and program owners to establish and maintain a strong public health emergency management system. In some woredas, there is currently no supportive supervision from government health offices and health facilities. In addition, many respondents recommended implementing monitoring and evaluation for FETP-Frontline and the public health emergency activities.

*“To have a strong system of public health emergency management there shall be a strong support from government and partner sides.”* Woreda Health Office, Afar Region.

## Discussion

Our evaluation identified perspectives of program implementers towards Ethiopia’s FETP-Frontline. The program implementers, including the regional, sub-regional, and districts health managers shared positive perceptions towards the training program. They identified areas that have shown improvement because of the training. In addition, they shared that districts with trained surveillance officers reflected better knowledge, skills and performance in surveillance activities compared to districts with untrained officers. The respondents also stipulated challenges and recommendations on how to improve and strengthen the FETP-Frontline.

Areas that have shown improvement after the training included early detection of outbreaks, prompt response to emergencies, staff motivation, understanding roles and responsibilities, surveillance data quality, and providing training and orientations to health facilities and community health workers. They also identified bottlenecks to the success of the program, including shortage of budget and human resource, lack of transportation, poor mentorship, and lack of career and professional development. Other challenges general to the public health system in the country, such as shortage of medical equipment and supplies, political instability, and staff turnover were also identified as drawbacks to the program’s effectiveness. Suggestions for improvement included mobilizing additional resources by inviting more stakeholders, strengthening mentorship, continued monitoring from stakeholders, and additional refresher training to the graduates.

These findings are in line with other FETP evaluation studies. The FETP-Advanced program has supported outbreak investigation, surveillance system strengthening and data quality improvement in diverse settings globally [7, 18–30]. FETP graduates have also achieved effective emergency response to man-made and natural disasters [19, 31]. Similarly, evaluation of FETP-Advanced showed that the graduates had responded to pandemics, such as Ebola [32] and COVID-19 [33–35].

Evaluation of FETP-Frontline in other countries also documented the effectiveness of the program. Studies conducted in Guinea [36], Liberia [37], Zambia [22] and Kenya [38] documented that the trained officers had improved capacity. Effectiveness of FETP-Frontline has also been reported with regards to improved surveillance data, quality surveillance reports, case investigations, outbreak investigations, and surveillance system improvements.

The findings in this study also showed similar challenges to those the FETP-Advanced graduates faced. The two most common challenges in FETP training were lack of sustainable funding [19, 20] and low retention of the workforce [19, 39]. Recommendations to address these challenges include linking FETP with ministries, universities, and international funding organizations [40] and providing professional development opportunities for graduates, including additional training [22, 34, 41].

Our study had a few limitations. First, the evaluation had limited scope and didn’t include program evaluation attributes such as efficiency. Instead, the evaluation and interview guide centered around pre-identified themes of perception of respondents towards the program and challenges and recommendations. While we can draw conclusions on the positive perception of FETP-Frontline in strengthening workforce capacity in Ethiopia, further study might be required on the wider impact of the training program. Second, the perceptions of donors and other funding partners were not included. We therefore recommend further research on costing and stakeholders’ analysis to determine sustainability of the program.

## Conclusion

FETP-Frontline has acceptability among implementing partners at various levels of the Ethiopian health care system. Our results showed that the program has improved knowledge and skills of district level surveillance officers in a wide range of surveillance activities, including data quality improvement, early detection and prompt response to outbreaks, reporting, communication, and provision of on-site orientation to health facility and community surveillance workers. While we recommend scaling-up of the training to reach more districts in the long-term, addressing immediate challenges such as transportation, supplies, and mentorship activities could be considered through mobilization of adequate resources. Further training and career development for FETP-Frontline graduates also require due attention.

## Data Availability

Data and other materials can be made available by the corresponding author upon a reasonable request.

## Abbreviations

CDC: US Centers for Disease Control and Prevention
EPHI: Ethiopian Public Health Institute
FETP: Field Epidemiology Training Program
IHR: International Health Regulations
PHEM: Public Health Emergency Management
SNNPR: Southern Nations, Nationalities and Peoples’ Region
WHO: World Health Organization
